# Search for Novel Therapies for Essential Tremor Based on Positive Modulation of α6-Containing GABA_A_ Receptors

**DOI:** 10.5334/tohm.796

**Published:** 2023-10-23

**Authors:** Adrian Handforth, Ram P. Singh, Marco Treven, Margot Ernst

**Affiliations:** 1 Neurology Service, Veterans Affairs Greater Los Angeles Healthcare System, Los Angeles, California, United States of America; 2 Research Service, Veterans Affairs Greater Los Angeles Healthcare System, Los Angeles, California, United States of America; 3 Department of Neurology, Medical Neuroscience Cluster, Medical University of Vienna, Vienna, Austria; 4 Department of Pathobiology of the Nervous System, Center for Brain Research, Medical University of Vienna, Vienna, Austria

**Keywords:** tremor, cerebellum, GABA_A_ receptors, harmaline

## Abstract

**Background:**

Prior work using GABA_A_ receptor subunit knockouts and the harmaline model has indicated that low-dose alcohol, gaboxadol, and ganaxolone suppress tremor via α6βδ GABA_A_ receptors. This suggests that drugs specifically enhancing the action of α6βδ or α6βγ2 GABA_A_ receptors, both predominantly expressed on cerebellar granule cells, would be effective against tremor. We thus examined three drugs described by *in vitro* studies as selective α6βδ (ketamine) or α6βγ2 (Compound 6, flumazenil) receptor modulators.

**Methods:**

In the first step of evaluation, the maximal dose was sought at which 6/6 mice pass straight wire testing, a sensitive test for psychomotor impairment. Only non-impairing doses were used to evaluate for anti-tremor efficacy in the harmaline model, which was assessed in wildtype and α6 subunit knockout littermates.

**Results:**

Ketamine, in maximally tolerated doses of 2.0 and 3.5 mg/kg had minimal effect on harmaline tremor in both genotypes. Compound 6, at well-tolerated doses of 1–10 mg/kg, effectively suppressed tremor in both genotypes. Flumazenil suppressed tremor in wildtype mice at doses (0.015–0.05 mg/kg) far lower than those causing straight wire impairment, and did not suppress tremor in α6 knockout mice.

**Discussion:**

Modulators of α6βδ and α6βγ2 GABA_A_ receptors warrant attention for novel therapies as they are anticipated to be effective and well-tolerated. Ketamine likely failed to attain α6βδ-active levels. Compound 6 is an attractive candidate, but further study is needed to clarify its mechanism of action. The flumazenil results provide proof of principle that targeting α6βγ2 receptors represents a worthy strategy for developing essential tremor therapies.

**Highlights:**

We tested for harmaline tremor suppression drugs previously described as *in vitro* α6βδ or α6βγ2 GABA_A_ receptor-selective modulators. Well-tolerated flumazenil doses suppressed tremor in α6-wildtype but not α6-knockout mice. Compound 6 and ketamine failed to display this profile, likely from off-target effects. Selective α6 modulators hold promise as tremor therapy.

## Introduction

A large fraction of GABA_A_ receptors is composed of two α (of which there are 6 types), two β subunits (3 types), and a δ (one type) or a γ subunit (3 types). Most brain GABA_A_ receptors incorporate γ, and are synaptic, exerting phasic inhibition in response to GABA. Those that contain δ are extra-synaptic, and exert tonic inhibition. In the latter receptors, δ is usually associated with α4 subunits throughout the brain, but in cerebellar granule cell (CGC) GABA_A_ receptors, α6 is the associated partner and is intensely expressed here, whereas α4 levels in the cerebellum are much lower, being expressed in the Purkinje cell (PC) layer and the molecular layer in mice [1,2]. CGCs from α6 knockout (KO, α*6^–/–^*) mice lack GABA-mediated tonic inhibition [3]. The location of α6βδ receptors on CGCs, where they respond to GABA released by Golgi neurons, provides a mechanism for controlling the excitatory CGC drive to PCs, which in turn are the sole output neurons for the cerebellar cortex.

The identification of new therapies for essential tremor (ET) has lagged, in part due to lack of known molecular targets. One strategy to address this problem is to “reverse engineer” a substance, such as alcohol, that reduces tremor in some ET cases, to identify molecular targets, then find more specific ligands for these targets with the goal of creating new drugs with better efficacy and tolerability. We previously found that low-dose alcohol suppresses tremor in the harmaline model of ET in wildtype (WT) mice, but fails to do so in KO littermates lacking either the δ or the α6 subunit [4]. Moreover, we found that ganaxolone and gaboxadol, which activate extra-synaptic GABA_A_ receptors allosterically or directly respectively, also each suppresses harmaline tremor, but not if either the δ or α6 subunit is lacking [4,5].

These observations with alcohol, well-known to suppress tremor in ET patients in low doses, are consistent with findings that low concentrations of alcohol enhance GABA-mediated currents in *Xenopus* oocytes expressing recombinant α6βδ receptors, and tonic currents in CGC slices, but not if the slices are taken from δ^–/–^ mice [6,7,8,9]. Given the predominant location of α6βδ GABA_A_ receptors on CGCs, these observations are also consistent with the finding from high-density electroencephalography (EEG) in ET subjects that tremor reduction by alcohol correlates with alterations of cerebellar activity [10], and with a blood flow study finding that a low dose of alcohol reduces the cerebellar hypermetabolism that occurs in ET towards normal levels [11,12], an observation that suggests that drugs that do this selectively should be well-tolerated, as it is a normalizing physiological action that suppresses tremor.

Within the cerebellum, α6 expression is virtually limited to the CGC layer [2]. Given the intense and relatively selective expression of α6βδ receptors on CGCs, they represent a promising target for ET therapy. Unfortunately, alcohol, ganaxolone, and gaboxadol also act on α4βδ GABA_A_ receptors that are expressed throughout much of the brain, causing unwanted effects; such effects may limit the dosage of these agents that can be administered, in turn limiting the maximal efficacy that can be achieved. For example, gaboxadol at 10 mg/kg causes sedation, impaired rotarod performance, and increases tail flick latency, but not if the mice are α4 KOs, although in these mice the drug is free to act on α6βδ GABA_A_ receptors [13]. These observations suggest that a drug that selectively activates or positively modulates α6βδ receptors without involving α4βδ GABA_A_ receptors should be well tolerated, permitting robust anti-tremor efficacy. A search for drugs that enhance α6βδ receptor action selectively is justified. In addition, as CGCs express α6βγ2 GABA_A_ receptors intensely [1], these represent an additional, potentially attractive target. Outside the cerebellum, α6 is expressed in the trigeminal ganglion [14], cochlear nuclei [15,16], and faintly in the spinal trigeminal nucleus [2]. This limited extra-cerebellar localization also suggests that selective modulation of α6 GABA_A_ receptors should be well-tolerated.

In a search for α6-selective drugs, we examined low-dose ketamine, Compound 6, and flumazenil. Ketamine has been reported to selectively enhance GABA-induced currents via *Xenopus* oocyte-expressed α6β2δ receptors, and to enhance tonic currents in CGCs in slices, but not if the slices are taken from α6- or δ-subunit KO mice [17]. Low-dose ketamine has been reported to ameliorate Parkinson’s tremor [18]. Ketamine has significant limitations in that it is an anesthetic that has other receptor actions, nonetheless we wondered whether low doses could suppress tremor through a selective α6 GABA_A_ receptor action.

Compound 6 (PZ-II-029) is a pyrazoloquinolinone, a class of compounds that exert very little sedation [19]. In oocyte recombinant systems, Compound 6 binds to an extracellular α-β site of αβ3γ2 receptors, displaying high selectivity for the α6 subunit among α subtypes. It also modulates αβ2γ2 receptors to a lesser degree [20,21]. In addition, it binds silently to the α-γ benzodiazepine binding site. Intraperitoneal (i.p.) injection of Compound 6 in mice blocks the disruption of prepulse inhibition caused by methamphetamine, whereas this effect of Compound 6 is prevented by intracerebellar injection of furosemide, which blocks α6 GABA_A_ receptors [22], suggesting that Compound 6 modulates α6βγ2 receptors *in vivo*. Limitations are that it has not been studied in receptor subunit KO mice or tissue, and the extent of non-GABA_A_ receptor brain binding sites has not been fully delineated. Given this lack of knowledge, there is a potential for binding to non-GABA_A_ receptor sites to interfere with testing the hypothesis of α6-dependent tremor suppression.

Flumazenil is marketed for reversal of benzodiazepine sedation, an action that is due to its silent binding at the α-γ benzodiazepine binding site within αβγ2 receptors. Flumazenil is also a partial positive modulator with some α-subunit selectivity. It enhances α6βγ2, α3βγ2, and α2βγ2 GABA_A_ receptor action *in vitro* at physiologically relevant levels, but not α1βγ2 receptors [23]. In humans, intravenous flumazenil, 1 mg, reduces anxiety [24] and improves finger tapping speed in Parkinson’s subjects [25], yet does not affect psychomotor performance at 0.1 mg/kg [26] or affect the EEG after 10 mg given intravenously [27]. This profile suggests that if flumazenil exerts α6-mediated tremor suppression in ET, this action may be well-tolerated. Unlike Compound 6, it is not a pyrazoloquinolinone, so that any binding to non-GABA receptor sites will differ. A potential limitation is that its positive modulation is only partial, so that maximal efficacy may not be marked.

In the present study, we sought to test the hypothesis that drugs that selectively or preferentially modulate α6-containing GABA_A_ receptors *in vitro* will exert anti-tremor effects in the harmaline model *in vivo* at well-tolerated doses, and in α6-dependent fashion, as assessed by comparing results from WT mice with those from α6 KO littermates. (The term “WT mice” is meant to indicate they possess normal copies of the gene that is deleted in KO mice.) It should be noted that in contrast to our earlier studies with alcohol, ganaxolone, and gaboxadol [4,5], none of the three candidate drugs in the present study have had prior supportive genetic GABA_A_ receptor animal behavior studies. Insofar as many drugs bind to multiple, often unknown, sites, binding to a non-GABA_A_ receptor site may lead to failure to translate from *in vitro* α6 activity to α6-dependent anti-tremor efficacy. For such anti-tremor efficacy to occur, each candidate drug had to pass two tests. In *Test 1*, the drug had to suppress tremor meaningfully in doses not associated with psychomotor impairment. In *Test 2*, anti-tremor efficacy that is demonstrable in WT mice must be abolished in α6 KO mice. Failures may occur because non-GABA_A_ receptor binding induces psychomotor impairment that prevents administration of doses sufficient to affect tremor, such as may happen if levels are too low to modulate α6 receptors (Test 1 failure), or because activation of a non-α6 GABA_A_ receptor site suppresses tremor in α6 KO mice (Test 2 failure). On the other hand, if a given drug passes both Test 1 and Test 2, so that it suppresses tremor in doses that do not cause psychomotor impairment, an effect abolished in α6 KO mice, then such a finding will support the hypothesis that an α6-GABA_A_ receptor modulating drug can suppress tremor in tolerated doses.

## Methods

### Study design

We evaluated each drug in a two-step process. In the first, we sought to determine the maximum dose at which 6/6 mice pass the straight wire test, a sensitive test of psychomotor impairment [28]. For example, we previously found that the maximum dose of alcohol at which 6/6 mice pass is 0.575 g/kg, a dose estimated to produce a blood level that is below the driving level limit of 0.080 g/dL [4]. Only a dose associated with 6/6 mice passing the straight wire test or lower doses were used in step two: effect of drug on harmaline tremor. Each drug was assessed for its ability to suppress tremor in WT mice and in littermates lacking the α6 receptor subunit. If a drug were to suppress tremor in WT but not in α6 KO mice, that would suggest the drug suppresses tremor by activating or modulating α6-containing GABA_A_ receptors. Each mouse received any drug or harmaline only once.

The harmaline model of ET was utilized. Harmaline elicits tremor by driving rhythmic, coupled inferior olivary bursting [29]. It is a symptom model, in which the brain areas activated during harmaline tremor overlap with the tremor circuit revealed by magnetoencephalography in ET [29,30,31]. This extensive circuitry overlap is consistent with considerable pharmacologic overlap, in which many drugs exert similar actions on ET and harmaline tremor [32].

Mice were assigned randomly to dosing groups, and the quantitation was performed by automated software. Animal protocols conformed to the National Institute of Health’s Guide for the Care and Use of Laboratory Animals (Eighth Edition, Washington DC, from the National Research Council, published in 2011), and were approved by the Veterans Affairs Greater Los Angeles Institutional Animal Care and Use Committee. All efforts were made to minimize animal suffering and to reduce the number of animals used.

### Animals

α6 KO (α*6^–/–^, Gabra6^–/–^*) mice were obtained from Jackson Laboratories (strain B6;129-*Gabra6^tm1Geh^*/J, #002710, Bar Harbor, ME). These had been generated with a 129 × 1/SvJ × 129S1/Sv cell line inserted into a C57BL6/J blastocyst [33] and were backcrossed with δ*^+/+^* mice in our laboratory for 10 generations; the δ*^+/+^* mice had been backcrossed with C57BL/6J for 11 generations as previously described [4]. Heterozygote mice were interbred to produce offspring that were genotyped with polymerase chain reaction (Transnetyx, Memphis, TN) and α*6^+/+^*, α*6^–/–^* littermates used for experiments. Both sexes were used as adults, and mice had *ad libitum* access to food and water.

### Test procedures

*Straight wire testing*. In this test, a mouse is suspended by the front paws from a rigid wire; to pass it must stay on the wire at least 10 seconds and touch the wire with a hind paw within those 10 seconds, and do so on each test conducted at 10-minute intervals for one hour following drug administration. A given drug dose passed if all 6 of 6 tested mice passed all tests; the highest dose at which 6/6 mice passed was sought.

*Harmaline-induced tremor*. Each mouse was placed on an 8.1-cm diameter mesh on top of a 24.1-cm high cylinder that rested on a Convuls-1 Replacement Sensing Platform model 1335-1A (Columbus Instruments, Columbus, OH), fitted with a load sensor, connected to a Grass model P511 AC amplifier (Grass Instruments, West Warwick, RI) with 1 and 70 Hz filter settings. Digitally recorded motion power was analyzed using Spike2 software (Cambridge Electronic Design; UK) to perform Fourier transformation of the data into frequency spectra. Data were sampled at 128 Hz. As previously described, harmaline-induced tremor occurs at 9–16 Hz, creating a corresponding motion power peak on digital frequency spectra [34,35]. To control for tremor power changes due to activity level fluctuation, this tremor-associated motion power bandwidth was divided by background overall activity motion power to form the measure of analysis, *motion power percentage* (MPP): (9–16 Hz motion power)/(0.25–32 Hz motion power) × 100 [35]. The use of such a ratio reduces variation compared to measuring tremor-associated motion power alone [35]. Placing each mouse on an elevated exposed small platform with intermittent rest periods in the home cage promotes vigilance and sustained tremor during motion power accession.

Mice were acclimated to the platform, then 15 minutes of pre-harmaline baseline motion data collected, then harmaline, 20 mg/kg, administered subcutaneously as 4 ml/kg. Once tremor had developed, within 10 minutes, motion power was again assessed during a 15-minute epoch. Ketamine, Compound 6, flumazenil, or corresponding vehicle was then injected i.p., 10 ml/kg. Motion power accession was re-initiated 10 minutes after injection for five more 15-minute epochs on the elevated platform (E1 to E5), with intervening 5-minute rests in the home cage.

### Drugs

Harmaline (Sigma-Aldrich, St. Louis, MO) and ketamine (Tocris Bio-Techne, Minneapolis, MN) were dissolved in saline. Compound 6 (synthesized in the laboratory of Marko D. Mihovilovic, Vienna Technical University, Vienna, Austria as previously described [36]) was dissolved by sonicating and warming in a mixture of 85% distilled water, 14% propylene glycol, and 1% Tween 80 (Sigma-Aldrich). Flumazenil (Tocris) was dissolved with warming in 1.25% alcohol (Thermo Fisher, Canoga Park, CA), 5% cremophor (Sigma-Aldrich), 93.75% saline with a drop of Tween 80.

### Statistical analyses

Mean motion power percentage (MPP) values were compared using a repeated measure (mixed) analysis of variance (ANOVA) model. A repeated measure model is needed since the same animal is measured repeatedly across 7 time periods (baseline, H, E1, E2, E3, E4, E5). Residual errors were examined using normal quantile plots (not shown) to confirm that the errors have a normal distribution, as required by this parametric model. The Shapiro-Wilk test for normality also confirmed that the errors followed a normal distribution. The model-based means and pooled standard errors (SEs) were calculated as well as p values for dose comparisons at each genotype-receptor and time and p values for genotype-receptor comparisons at each dose and time. The p values were deemed significant using the Fisher least significant difference (Fisher LSD) criterion.

Computations were carried out using R 4.0.5 (R Foundation for Statistical Computing, Vienna, Austria, https://www.R-project.org/).

## Results

### Ketamine

The highest dose at which 6/6 passed all straight wire tests over an hour after ketamine administration was 3.5 mg/kg; at 3.75 mg/kg, not all 6 of 6 mice passed. Accordingly, the doses chosen for evaluation were 3.5 and 2.0 mg/kg.

**Figure 1:**
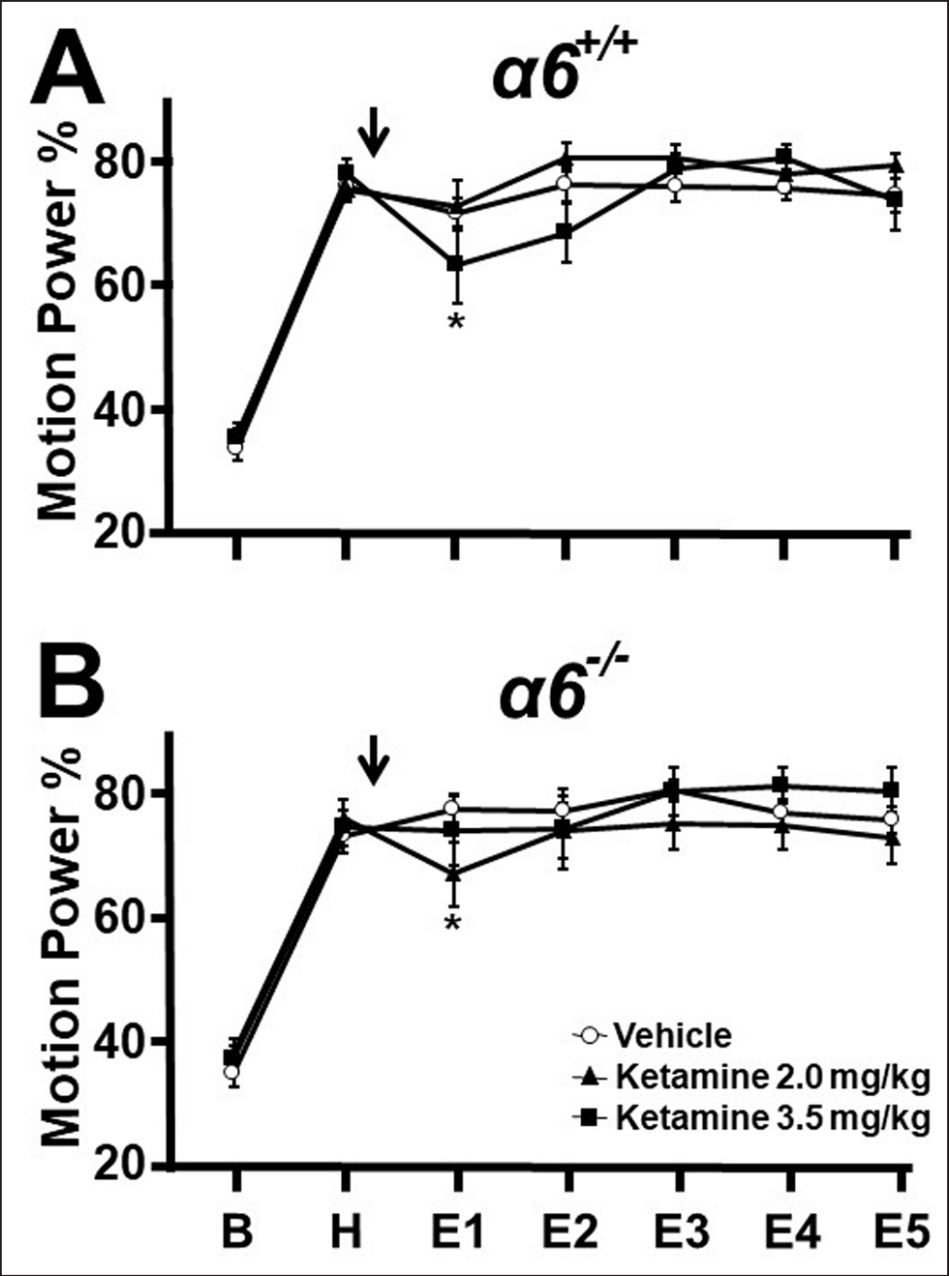
**Effect of ketamine on harmaline tremor in α*6*^+/+^ (α6 WT) and littermate α*6*^–/–^ (α6 KO) mice.** Motion power in groups of mice was followed sequentially during 15-minute epochs at baseline (B), pre-treatment harmaline (H), and after vehicle or ketamine injection (arrow, E1-E5). **A.** α*6^+/+^* **B.** α*6^-/-^* Ketamine in doses that did not cause psychomotor impairment on the straight wire test, 3.5 and 2.0 mg/kg, had minimal effect in both genotypes compared to vehicle controls. * p < 0.05, ** p < 0.01, *** p < 0.001, ANOVA with Fisher least significant difference criterion.

In α*6^+/+^* mice, the motion power percentage (MPP) that fell by chance within the 9–16 Hz bandwidth approximated 30–40% during the 15-minute pre-harmaline baseline (B) (Figure 1A). With harmaline administration, the MPP increased to 75–80%, so that tremor dominated motion power during the 15-minute harmaline pre-treatment epoch (H). Mice were then given vehicle or ketamine, 3.5 or 2.0 mg/kg i.p., n = 16 per group. After vehicle injection, harmaline tremor-associated elevated MPP was sustained for five more 15-minute epochs (Figure 1A). In comparison with the vehicle control group, ketamine at 3.5 mg/kg reduced tremor mildly in the first post-injection epoch (E1, vehicle vs 3.5 mg/kg means: 73.1 vs 64.6, p = 0.043), but not at 2.0 mg/kg (vehicle vs 2.0 mg/kg means: 73.1 vs 74.3, p = 0.782) or in post-injection epochs E2 to E5.

Littermate α*6 ^–/–^* mice are indistinguishable from α*6^+/+^* mice, and displayed pre-harmaline baseline and pre-treatment harmaline MPP values comparable to those of α*6^+/+^* mice, indicating no alteration in harmaline tremor response. Figure 1B displays motion power in 12, 10, 10 α*6 ^–/–^* mice receiving vehicle or ketamine 2.0 or 3.5 mg/kg. The dose 2.0 mg/kg caused mild suppression during E1 (vehicle vs 2.0 mg/kg means: 77.6 vs 67.0, p = 0.039) whereas 3.5 mg had no effect (vehicle vs 3.5 mg/kg means: 77.6 vs 74.1, p = 0.491). No effect was seen in subsequent post-injection epochs. These findings indicate that low doses of ketamine that avoid psychomotor impairment exert only minimal effects on tremor that are transient and likely not related to the presence of the α6 subunit. These results are interpreted as a Test 1 failure, in that off-target effects of drug that caused psychomotor impairment limited the testable dose to levels that did not induce meaningful tremor suppression.

### Compound 6

**Figure 2:**
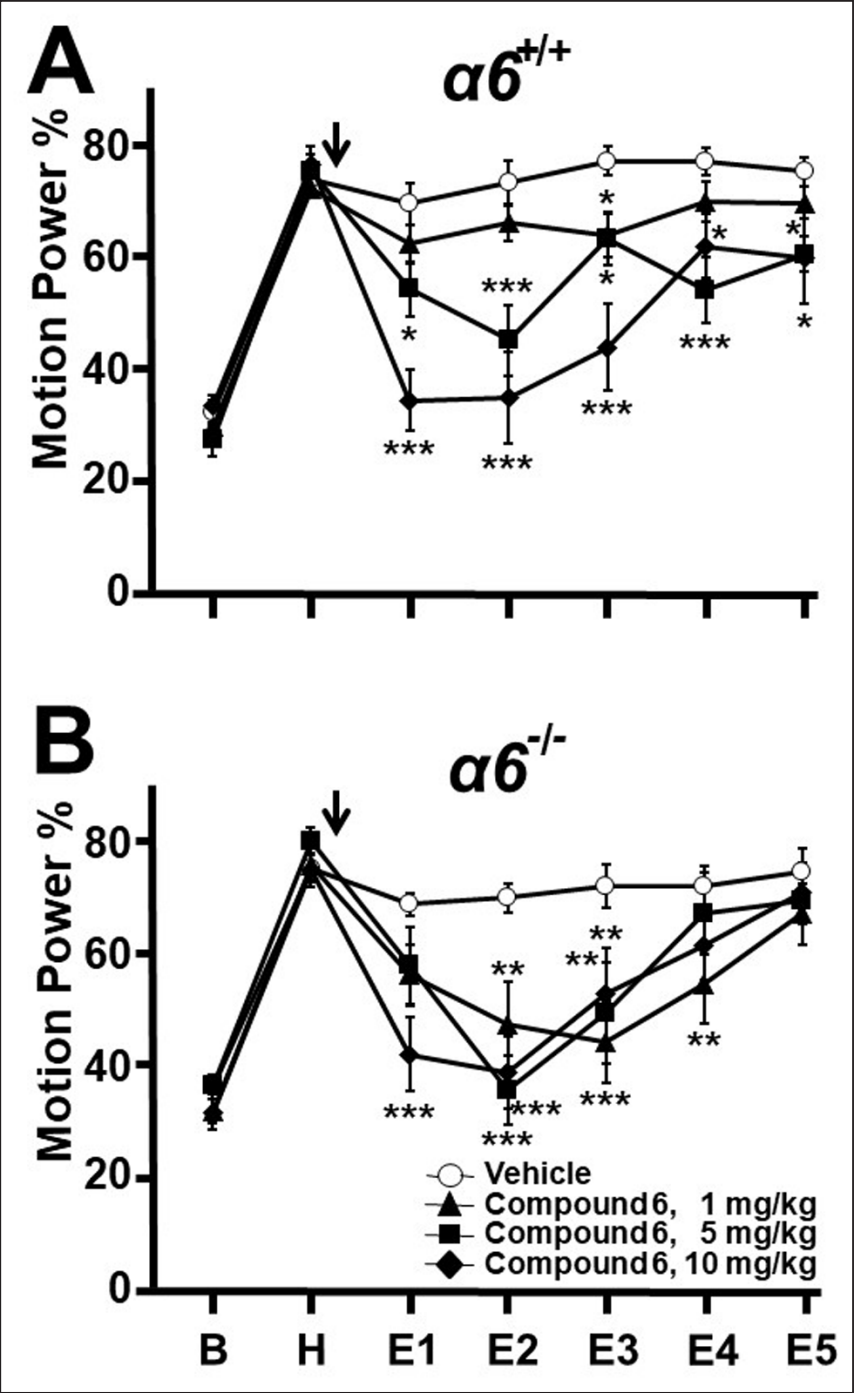
**Effect of Compound 6 on harmaline tremor in α*6*^+/+^ and littermate α*6*^–/–^ mice.** Motion power was followed sequentially during 15-minute epochs as described in Figure 1. **A.** α*6.^+/+^* Compound 6 in well tolerated doses suppressed tremor compared to vehicle controls. **B.** α*6.^-/-^* Despite lacking the α6 GABA_A_ receptor subunit, these mice also responded to Compound 6 with tremor reduction. * p < 0.05, ** p < 0.01, *** p < 0.001, ANOVA with Fisher least significant difference criterion.

At the highest dose tested, 20 mg/kg, all 6/6 α*6^+/+^* mice passed straight wire testing. Limited solubility required that this dose be given as 20 ml/kg. Higher doses could not be given as this would have exceeded permissible limits on injection volumes. Following the harmaline pre-treatment epoch (H), α*6^+/+^* mice were administered vehicle or Compound 6 in doses of 1, 5 and 10 mg/kg, all as 10 ml/kg (n = 16, 17, 11, 11 respectively). Compound 6 exerted tremor suppression that was dose-related (Figure 2A). At 10 mg/kg, Compound 6 suppressed tremor markedly, with effects seen in post-injection epochs E1 to E5 (vehicle vs 10 mg/kg means: 69.3 vs 34.0, p < 0.0001; 73.3 vs 34.4, p < 0.0001; 77.0 vs 43.5, p < 0.0001; 77.0 vs 61.7, p = 0.019; 75.1 vs 59.6, p = 0.039 respectively). At 5 mg/kg, tremor suppression was less marked but statistically significant from E1 to E5 (vehicle vs 5 mg/kg means: 69.3 vs 54.0, p = 0.019; 73.3 vs 44.8, p < 0.0001; 77.0 vs 62.9, p = 0.030; 77.0 vs 53.7, p < 0.0005; 75.1 vs 60.3, p = 0.036 respectively). At 1 mg/kg, mild tremor suppression occurred during E3 only (vehicle vs 1 mg/kg means; 77.0 vs 63.7, p = 0.022).

Littermate α*6^–/–^* mice received vehicle or Compound 6 in doses of 1, 5, 10 mg/kg (n = 11, 14, 11, 11). Although prior *in vitro* studies indicated a selective α*6* action, α*6^–/–^* mice receiving Compound 6 displayed significant tremor suppression (Figure 2B). The dose 10 mg/kg suppressed tremor markedly in E1 to E3 (vehicle vs 10 mg/kg means: 68.6 vs 42.0, p = 0.0002; 69.6 vs 38.8, p < 0.0001; 71.7 vs 52.8, p = 0.0079 respectively), while 5 mg/kg reduced tremor less markedly in E2 and E3 (vehicle vs 5 mg/kg means: 69.6 vs 35.6, p < 0.0001; 71.7 vs 49.3, p = 10.0018 respectively), while 1 mg/kg suppressed tremor in E2 to E4 (vehicle vs 1 mg/kg means: 69.6 vs 47.2, p = 0.0010; 71.7 vs 44.3, p < 0.0001; 71.7 vs 54.3, p = 0.0099 respectively). In between-genotype comparisons, the MPP values for each epoch E1 to E5 did not differ significantly between KO and WT mice at 5 and 10 mg/kg, but were lower during E2 to E4 at 1 mg/kg in KO mice (KO vs WT means: 47.2 vs 65.9, p = 0.0021; 44.3 vs 63.7, p = 0.0014; 54.3 vs 69.7, p = 0.0104 respectively). These results indicate that in α*6^–^^/–^* mice, Compound 6 at 5 and 10 mg/kg suppresses tremor to a degree comparable to that seen in α*6^+/+^* mice, but at 1 mg/kg exerts more tremor suppression in the KO mice. The failure to show abolition of tremor suppression in KO mice represents a Test 2 failure, in that specificity of the anti-tremor action to the α6 receptor was not demonstrated.

### Flumazenil

**Figure 3:**
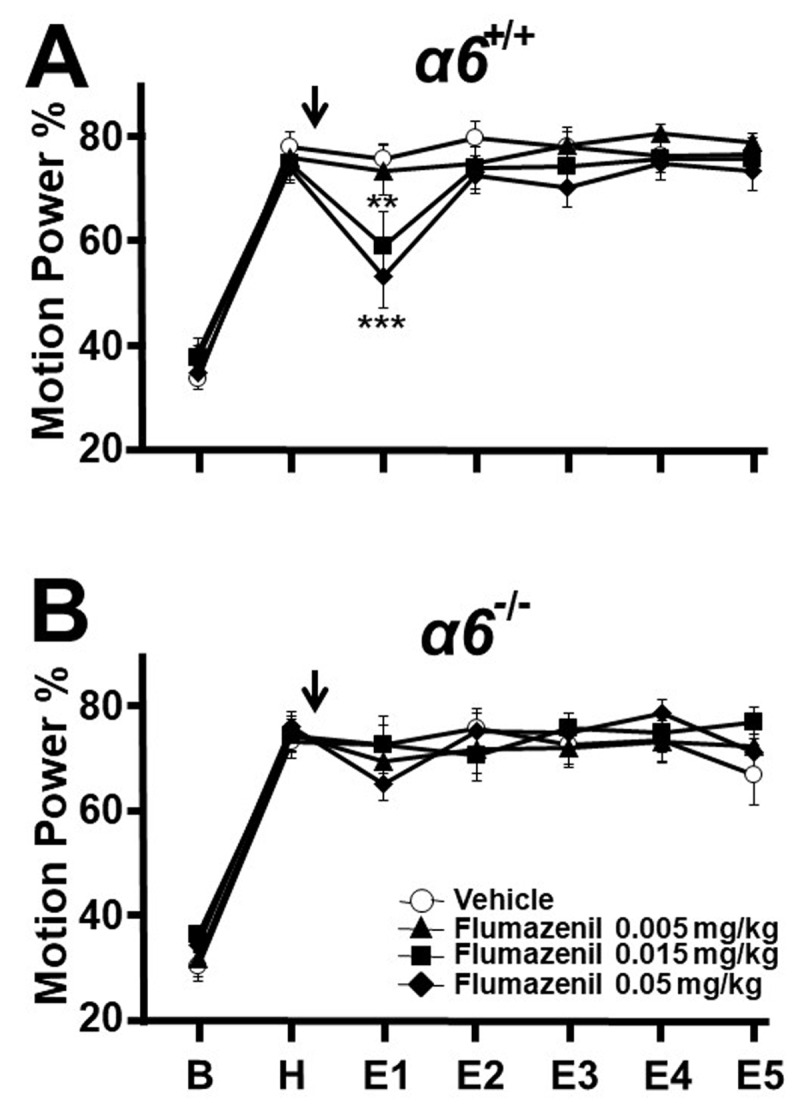
**Effect of flumazenil on harmaline tremor in α*6*^+/+^ and littermate α*6*^–/–^ mice.** Motion power was followed sequentially during 15-minute epochs as in Figure 1. **A.** α*6.^+/+^* Flumazenil in well tolerated doses suppressed tremor dose-dependently compared to vehicle controls. **B.** α*6.^-/-^* Littermates lacking the α6 GABA_A_ receptor subunit did not respond to flumazenil with tremor reduction. * p < 0.05, ** p < 0.01, *** p < 0.001, ANOVA with Fisher least significant difference criterion.

**Figure 4:**
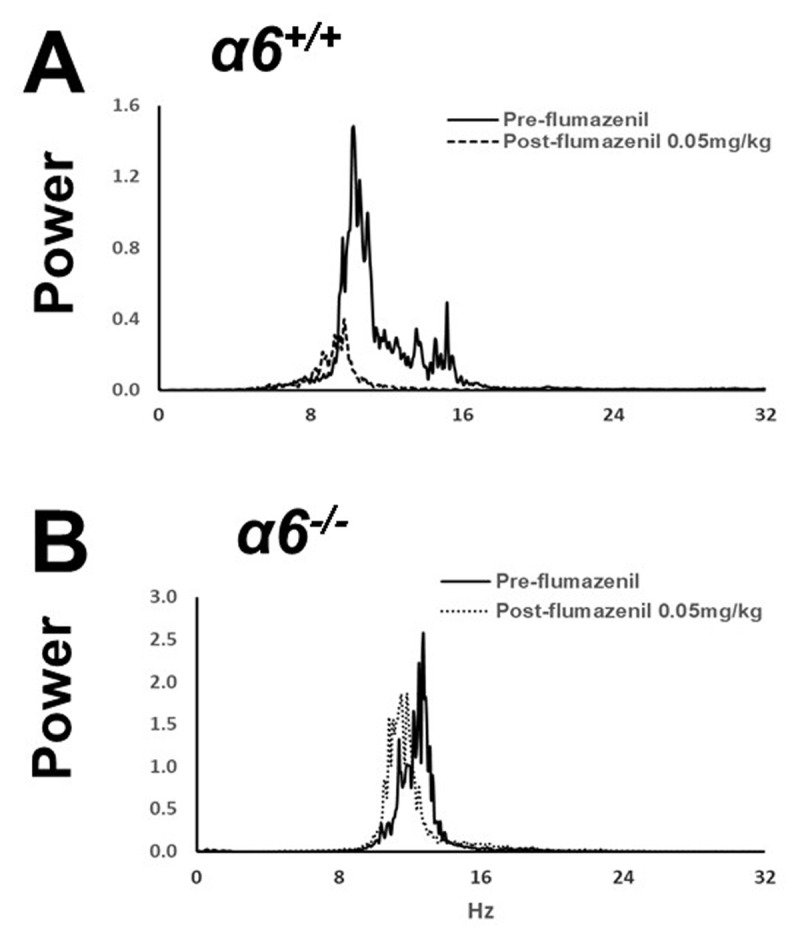
**Examples of motion power spectra of harmaline tremor in α*6*^+/+^ and α*6*^–/–^ mice before and after receiving flumazenil, 0.05 mg/kg i.p. A.** α*6^+/^*^+^ In this example, the dominant motion power peak corresponds to harmaline-induced tremor. This peak is reduced upon administration of flumazenil. **B.** α*6^–/–^* This example shows a comparable harmaline-associated motion power peak. In the absence of the α6 subunit, flumazenil had little effect on this motion power peak.

At 112.5 mg/kg, all 6/6 α*6^+/+^* mice passed straight wire testing, whereas not all passed at 125 mg/kg. Pilot testing indicated that far lower doses were associated with tremor reduction. In harmaline tremor experiments, α*6^+/+^* mice were injected with vehicle or flumazenil, 0.005, 0.015, or 0.05 mg/kg (n = 12, 10, 11, 12). Any effect on tremor was expected only in post-injection epoch E1, as flumazenil is cleared rapidly. It was found that flumazenil displayed dose-dependent tremor suppression, with 0.05 and 0.015 mg/kg moderately suppressing tremor during E1 (vehicle vs dose means: 75.6 vs 53.1, p < 0.0001; 75.6 vs 59.0, p = 0.0016 respectively), while 0.005 mg/kg had no significant effect (Figure 3A). Tremor during subsequent post-injection epochs was not affected by flumazenil. A display of motion power according to motion frequency is shown in Figure 4A for an example α*6^+/+^* mouse during harmaline tremor before and after treatment with flumazenil, 0.05 mg/kg, in E1 and demonstrates suppression of the motion power peak associated with tremor.

When α*6^–/–^* littermate mice were similarly treated with vehicle or flumazenil, 0.005, 0.015, or 0.05 mg/kg (n = 12, 12, 12, 14), there was no significant reduction of tremor during E1 or other epochs at any dose (Figures 3B, 4B), indicating that flumazenil requires the presence of α6-containing GABA_A_ receptors to exert tremor suppression. In summary, flumazenil passed both Test 1 and Test 2, displaying anti-tremor efficacy at well-tolerated doses and a requirement for α6 GABA_A_ receptors. In light of prior evidence that flumazenil exerts modulation of α6 GABA_A_ receptors *in vitro*, these findings support the hypothesis that drugs with such a profile may suppress tremor in well-tolerated doses.

## Discussion

We previously found that gaboxadol, low-dose ethanol, and the neurosteroid ganaxolone each suppresses harmaline tremor in doses that do not cause impairment on the straight wire test, but had no effect on tremor in δ KO or in α6 KO mice [4,5], suggesting that these drugs acted on α6βδ GABA_A_ receptors, most likely on CGCs, where they are intensely expressed. However, the concurrent activation or modulation of α4βδ GABA_A_ receptors that are expressed in numerous brain sites outside the cerebellum compromises the potential utility of these agents, as their enhanced action would induce unwanted effects. For example, doses of gaboxadol higher than 3 mg/kg could not be tested on tremor, as they caused impairment on straight wire testing [5]. Doses of 4–5 mg/kg affect the EEG in mice, but not in δ KO mice [37], an effect likely mediated by α4βδ GABA_A_ receptors in the cerebral cortex.

We considered low-dose ketamine as a candidate drug of interest. Hevers et al [17]. reported that ketamine enhances GABA-induced currents by α6β2/3δ receptors, with specificity for the α6, β2, β3, and δ subunits. Moreover, they found that ketamine induced tonic currents in CGCs, an action that depended on α6 and δ subunits [17]. Ketamine’s most-studied action is blockade of N-methyl-D-aspartate (NMDA) receptors [38,39]. However, Hevers et al. argued that the action of ketamine on CGCs may be clinically relevant, citing evidence that modulation of GABA_A_ receptors occurs at levels below those that block NMDA receptors [17]. We therefore examined whether subanesthetic, non-impairing doses could suppress tremor, and do so in an α6-selective fashion. We found that the highest dose passed by all WT mice on the straight wire test was 3.5 mg/kg. At this dose or at 2.0 mg/kg there was only minimal and transient suppression of harmaline tremor in α6 WT and KO littermates. Ketamine has multiple actions, as summarized by Hevers et al [17] and it appears that these effects permitted only very low doses to be assessed that do not cause psychomotor impairment. Based on a pharmacokinetic study of ketamine administered to mice [40], it can be estimated that a dose of 3.5 mg/kg i.p. will result in a brain level of 1.6 µM, well below the range of 10–100 µM found by Hevers et al [17]. to enhance GABA currents via α6β2δ receptors in oocytes. The lack of efficacy by ketamine thus represents a Test 1 failure. Nonetheless, these receptors remain an inviting therapeutic target awaiting a selective positive allosteric modulator.

Pyrazoloquinolinones have been investigated as potential anxiolytics with an exceptionally low potential for sedation [19]. Certain members of this class are functionally selective for α6β2/3γ2 receptors in recombinant systems, including Compound 6 [21]. It binds silently to the α-γ benzodiazepine site, consistent with the finding that, at well-tolerated doses, it blocks the ataxic effect of the benzodiazepine diazepam in rats [41]. Through its binding to an extracellular α-β site on αβ3γ2 receptors, it is highly selective for the α6 subunit over other α subunit types. It also modulates αβ2γ2 receptors to a lesser degree [21]. A cerebellar site of action has been inferred by Chiou et al., [22] who showed that intracerebellar injection of the α6-containing receptor antagonist furosemide prevents systemically administered Compound 6 (3 or 10 mg/kg) from blocking methamphetamine-induced prepulse inhibition disruption. A deuterated analog of Compound 6 has been found effective in a rat model of trigeminal neuralgia [42], possibly due to modulation of α6βγ2 GABA_A_ receptors located in certain cells of the trigeminal ganglion [14]. Comparison of results with α6 KO mice has not previously been described.

Due to limitations of solubility and permissible volumes of injection, 20 mg/kg was the highest dose that could be tested on straight wire testing; at this dose all 6/6 mice passed. Thus the dose that causes psychomotor impairment is not known, but is more than 20 mg/kg in mice. In harmaline tremor studies with WT mice, Compound 6 exerted dose-dependent tremor suppression at 1–10 mg/kg. These doses are comparable to those employed by Chiou et al [22]. We found that α6 KO mice displayed comparable tremor suppression at 5 and 10 mg/kg and more tremor suppression than WT mice at 1 mg/kg. In interpreting these results, a consideration is whether Compound 6 might affect the compensating changes present in α6 KO mice. In these mice, 50% of cerebellar GABA_A_ receptors are lost, including both synaptic and extra-synaptic receptors [43,44]. Yet α6 KO mice exhibit no motor deficits [43,44], and have normal harmaline tremor. Voltage-independent potassium conductance is upregulated in CGCs of these mice, appearing to underlie at least part of the compensation [3]. Other, unknown, compensations may also be present in α6 KO mice.

In summary, the results with Compound 6 represent an instance of Test 2 failure, in which α6 KO mice have failed to lose the tremor suppression seen in WT littermates, so that the drug cannot be concluded to suppress tremor by an α6-dependent action. This may be secondary to an action by Compound 6 on a non-GABA_A_ receptor target, either in α6 KO mice alone, or in both WT and KO mice. Further work is needed to understand Compound 6’s *in vivo* mechanism of action, but its high anti-tremor efficacy at well-tolerated doses suggests that this compound and others of its class hold promise for ET.

Flumazenil is a near silent binder with antagonistic effects at diazepam sensitive (DS) α-γ benzodiazepine binding sites, and acts as a positive GABA_A_ receptor modulator in diazepam-insensitive α6β3γ2 receptors [23]. In humans, it exerts various therapeutic effects in doses well below those associated with impairment [24,25,26,27]. Flumazenil is short-acting, with an elimination half-life in humans of 70 minutes [27].

We found that 112.5 mg/kg was the highest dose passed on the straight wire test by 6/6 mice. In harmaline experiments, doses of 0.05 and 0.015 mg/kg were found to suppress tremor in WT mice during the first post-injection epoch. In contrast, α6 KO mice displayed no tremor suppression in response to flumazenil. These results suggest that flumazenil suppresses tremor by modulating α6βγ2 GABA_A_ receptors on CGCs, and does so with striking tolerability, so that doses 2000 times higher than anti-tremor doses are required to cause psychomotor impairment. Although flumazenil is a positive modulator of α2βγ2 and α3βγ2 GABA_A_ receptors [23], CGCs do not express these receptors [1], and flumazenil lacks activity for α1βγ2 receptors [23], so that the ability of this drug to suppress tremor in WT but not in α6 KO mice is consistent with an action on CGC α6βγ2 receptors.

In summary, flumazenil passed Tests 1 and 2, displaying tremor suppression in highly tolerated doses in WT mice, but not in α6 KO littermates. These findings, combined with the *in vitro* evidence, support the hypothesis that α6 GABA_A_ receptor-selective medications can suppress tremor effectively in well-tolerated doses, providing that off-target binding does not interfere. Flumazenil is the fourth drug that we have found to suppress tremor in α6-dependent fashion, the others being low-dose alcohol, ganaxolone, and gaboxadol [4,5], but the latter three modulate α6βδ and also α4βδ GABA_A_ receptors, so that achievable doses are limited by psychomotor effects, whereas flumazenil’s relative selective action on α6βγ2 receptors enables a favorable tolerability/efficacy profile. Flumazenil is on the market, but its rapid clearance would require that a prolonged delivery system be used or the compound undergo molecular modification to slow clearance if it were to be used as ET therapy. Another potential limitation is that in the harmaline model, tremor suppression was only moderate, likely due to a positive allosteric modulatory effect on α6βγ2 GABA_A_ receptors that is partial [23]. Thus a compound with stronger positive modulation at α6βγ2 receptors, and longer duration of action, may possess greater anti-tremor efficacy. It may also be noted that patients treated chronically with flumazenil (or Compound 6) could not be administered benzodiazepines due to the blockade of the GABA_A_ receptor benzodiazepine binding site by these drugs.

How would positive modulation of CGC α6 GABA_A_ receptors suppress tremor? As summarized in a review [29], it is postulated that the level of CGC parallel fiber-induced PC simple spike (SS) activity affects the synchrony of PC complex spike (CS) firing, deep cerebellar nucleus (DCN) burst-firing synchrony, downstream thalamic synchrony, and thus tremor. CSs are spike bursts triggered at inferior olivary (IO) climbing fiber synapses on PCs [45]. CSs among multiple PCs are synchronized by coupled clusters of projecting IO neurons [46], so that the degree of PC CS synchrony is controlled by the degree of IO coupling. The convergent action of synchronized PC CSs potently inhibits DCN neurons [47,48], provoking hyperpolarization-induced rebound bursting [49] that is transmitted to the thalamus; thus the degree of PC CS synchrony is important for movement amplitude and tremor. When IO coupling is increased by local injection of the GABA_A_ receptor antagonist picrotoxin, increased PC CS synchrony and increased movement amplitude ensues [50]; and, in some animals, tremor occurs [51]. Likewise, systemic harmaline and intra-IO serotonin receptor 2a agonists increase IO coupling [52,53,54], increase PC CS synchrony [54,55], and induce tremor [53,56]. Larger ensembles of IO increase PC CS synchrony [54,55], and induce tremor [53,56]. In contrast, intra-IO GABA release inhibits coupling, thereby reducing PC CS synchrony [51,57]. The main source of GABA in the IO is the massive GABAergic projection from DCN [58]. These IO-projecting DCN neurons in turn are inhibited by GABA released by PC terminals as PCs engage in SS activity [59,60]. These structures thus form a tri-synaptic circuit, in which PCs that respond with SSs to CGC parallel fibers project GABAergic fibers to DCN neurons that in turn project GABAergic fibers to IO neurons that control PC CS synchrony within the same territory affected by parallel fiber input. Application of the GABA_A_ receptor agonist muscimol to rat cerebellar cortex reduces PC SS firing, disinhibiting DCN neurons so that they release more GABA within IO, reducing coupling and therefore PC CS synchrony [61]. As we postulate that excess PC CS synchrony may be associated with tremor [28], such an action, as with a modulatory drug action on α6β3δ or α6β3γ2 GABA_A_ receptors on CGCs, would be to exert anti-tremor effects. This conceptualization posits that increased PC CS synchrony underlies tremor. That this is the case is suggested by the finding that ET cases display cerebellar oscillations in field potential recordings [62]. These also occur in the *hotfoot17* mouse model, which displays pathologic features of ET, and are synchronous with olivary firing [62]. The *hotfoot17* and harmaline models of tremor share a number of features, including the required integrity of IO, climbing fiber-PC synapses, and of PC-DCN synapses [29,62].

A potential limitation in this work is that we did not confirm the lack of α6 gene product in α6 KO mice with Western blot. However, this strain has long been established [33], and in contrast to WT littermates these mice were found to lack tremor suppression in response to alcohol, gaboxadol, ganaxolone, and in the present experiments, to flumazenil. It is thus unlikely that the results seen with Compound 6 and ketamine in KO mice were due to preserved α6 expression.

Another potential limitation is that the harmaline model may potentially produce a false positive result. The circuit activated by harmaline matches the overall circuit activated in ET as revealed by magnetoencephalography [30], including DCN, thalamus, motor cortex, and cerebellar cortex (PCs, CGCs), and brainstem [11,29,31,63]. Thalamic deep brain stimulation suppresses harmaline tremor as well as tremor in ET [64]. The shared anatomy in the model and in ET is in concordance with a similar pharmacologic response profile [32]. Indeed, the harmaline model has successfully predicted clinical efficacy by 1-octanol [65,66], T-type calcium channel antagonists [34,67,68], and AMPA receptor antagonists [69,70] for ET. However, there is some non-overlap between ET and harmaline model circuitry, as some drugs affect harmaline tremor that are not clinically effective, such as dopaminergic drugs [32].

In conclusion, we examined three compounds for anti-tremor efficacy based on their described *in vitro* activity as positive modulators of α6 GABA_A_ receptors. The prediction that this approach would be fruitful in identifying new anti-tremor compounds was based on our previous findings that low-dose alcohol, ganaxolone, and gaboxadol depend on α6 GABA_A_ receptors for anti-tremor efficacy. However, *in vitro* activity on α6 receptors may fail to translate into an α6-dependent action on tremor if the tolerated dose is too low to affect α6 receptors and tremor (Test 1 failure), or if the compound acts on another, unknown target to affect tremor in non-α6-dependent fashion (Test 2 failure). Table 1 summarizes the present findings. Ketamine was tolerated only in low doses, so that brain levels likely did not achieve levels of 10–100 µM needed to modulate α6 receptors [16], leading to failure to display anti-tremor efficacy. An α6β2/3δ-selective positive modulator of GABA_A_ receptors remains to be identified, but is an attractive target for tremor suppression. Compound 6, a pyrazoloquinolinone, suppressed tremor in both α*6^+/+^* and α*6^–/–^* mice, and was thus a Test 2 failure by not showing an α6-dependent action. Nonetheless, it exerted robust tremor suppression at well-tolerated doses, suggesting it may have potential clinical utility. Flumazenil’s tremor suppression was α6-dependent, and displayed a very high behavioral toxicity to tremor efficacy ratio. The flumazenil findings support the hypothesis that an α6 GABA_A_ receptor-selective modulator may suppress tremor at well-tolerated doses, as long as its binding profile exhibits freedom from interfering effects of off-target binding. These results suggest that a continued search for GABA_A_ α6βδ- and α6βγ2-receptor-selective positive modulators as potential novel therapies for ET is justified.

**Table 1:** Outcomes of tremor testing in drugs displaying *in vitro* α6 GABA_A_ receptor selectivity.


DRUG	SUPPRESSED TREMOR WELL IN		PASSED		INTERPRETATION
	
	α*6^+/+^*	α*6^–/–^*	TEST1	TEST 2	

Ketamine	No	No	No	NA	Binding to non-GABA_A_ sites prevented testing of doses high enough to affect α6 receptors.

Compound 6	Yes	Yes	Yes	No	By suppressing tremor in α*6^–/–^* mice, it failed to display α6 selectivity, likely by binding to an unknown non-GABA_A_ receptor site.

Flumazenil	Yes	No	Yes	Yes	This result supports the hypothesis that a drug with α6 GABA_A_ receptor selectivity *in vitro* may suppress tremor with α6 selectivity.


## Data accessibility statements

Data are available from the corresponding author upon reasonable request.
